# A Practical Treatise on the Diseases Peculiar to Women; Illustrated by Cases Derived from Hospital and Private Practice

**Published:** 1841-01

**Authors:** 


### ART. X.

1. *A Practical Treatise on the Diseases peculiar to Women; illustrated by Cases derived from Hospital and Private Practice.* By SAMUEL ASHWELL, M.D., Obstetric Physician, and Lecturer to Guy's Hospital. Part I. *Functional Diseases.—London,* 1840. 8vo, pp. 208.

2. *A Practical Treatise on the Function and Diseases of the Unimpregnated Womb. Illustrated by Plates, &c.; with a Chapter on Leucorrhœa, Fluor Albus, or "Weakness."* By CHARLES WALLER, M.D., Lecturer on Midwifery at the Medical School, Aldersgate Street, &c. &c.—*London,* 1840. 8vo, pp. 200.

IT is quite true, as Dr. Ashwell observes in his preface, that although we possess many valuable single essays on female diseases, there is scarcely one complete and practical work. His aim has been to produce a treatise on this subject, "so true, simple, and practical, that it may form a safe and efficient guide to the elucidation and curative treatment of many, at least, of these intricate, rapidly-progressing, and dangerous maladies." He devotes his work strictly to pathology and treatment, not to anatomical detail and physiological research, and it includes little which is not the product of his own practice and observation. Many formulæ of remedies are appended to the various chapters, and this has been done because it harmonized with the practical plan he has adopted.—Dr. Waller's intention is less extensive than Dr. Ashwell's. He is anxious to present to the profession a condensed account of the most common diseases to which the uterine system is obnoxious.—Passing over the brief introductory remarks with which both the volumes begin, we shall briefly abstract and comment upon the views of the writers on the different diseases they treat of; and first of

CHLOROSIS. Dr. Ashwell's definition of this common and obstinate disease of females may be objected to on account of its length, but its general accuracy will, we think, be admitted. He defines chlorosis to be a peculiar affection of the general health, in which debility, languor, and deranged stomachic functions are prominent symptoms, most frequently occurring when puberty is or ought to be established, although it may exist at any subsequent period, always characterized by anæmia of the system, and a yellowish dirty green pallor of the surface: when a disease of early youth, almost invariably connected either with entire absence of menstruation, or with a scanty, painful, and irregular performance of the function; and if a disease of later life, in addition to these causes it may have been preceded and produced by menorrhagia or leucorrhœa. Chlorosis, Dr. Ashwell says, presents itself in three forms: as a mild and incipient, an inveterate and confirmed, and a complicated disease.* Now this, like all, or at least most nosological arrangements is quite an arbitrary one, simply because nature does not choose either in health or disease to be limited and confined by our abstract rules, but still we agree that these forms which are laid down by Dr. Ashwell are sufficiently comprehensive and correct to embrace the great majority of cases of chlorosis which we meet with in practice.

The incipient form of chlorosis commences almost unobserved: the patient has most likely been "delicate" from infancy, but at the age of twelve, thirteen, or fourteen years without any obviously exciting or morbid cause, other than a negative one, the non-establishment of puberty, a series of distressing and perplexing symptoms are ushered in. The general health declines in connexion either with the entire or partial absence of menstruation. The girl does not pass on to womanhood. She is languid, soon fatigued, dull, and listless, sometimes perverse and sullen, and prone to solitude. The appetite is capricious. The complexion is pale; bowels constipated; tongue of a dirty pasty white; the breath is offensive; flatulence; frequent and severe headachs; palpitation of the heart and pain in the side; quick, weak, and compressible pulse are common symptoms. The menses, if not absolutely retarded, have scarcely appeared, the discharge having been pale in colour, and scanty in quantity. These symptoms, (with, we would add, many variations in different cases that long observation alone can teach,) in an aggravated degree, and some new ones, attend inveterate and confirmed chlorosis. Complicated chlorosis consists in the presence, in the greater or less degree, of the general symptoms of the disease, with a more prominent morbid affec-

* Dr. Copland adopts the same distinctions of chlorotic cases. Dict. of Pract. Med. Part i. p. 316.—REV.

tion of some distant organ. The predisposing and exciting causes of the diseases are, in brief, such as tend to enfeeble the mental and bodily powers, and to interfere more or less with the functions of the ovaria and uterus, especially about the age of puberty.

In common with most writers of the day, Dr. Ashwell thinks it may fairly be assumed that chlorosis primarily depends on a morbid condition of the blood, which secondarily affects the ovaries and uterus by retarding their growth. This opinion is supported by the fact, that in the blood of chlorotic patients there is an increased proportion of the serum, with a marked diminution of the crassamentum. Gooch and Andral distinctly allude to the deteriorated quality of the blood. The former says* "if in girls who have attained the age at which this change is customary, the sexual organs are not developed, a deranged state of the constitution occurs, characterized by peculiar symptoms, &c. In addition, the quality of the circulating fluid is in chlorosis altered; blood has been taken by way of experiment, and it has been found to be of a pale red colour and watery like the juice of a cherry." Andral again confirms these views.† "If chlorosis, as is now generally admitted, frequently results from a defective formation of the blood, the cause of which may reside exclusively in the nervous system, can one with any show of reason refer either to irritation or sanguinary congestion, the Proteus-like variety of functional derangements which chlorotic patients so constantly present, such as epileptic paroxysms, convulsions, chorea, dyspnea, palpitations, vomiting &c.? Or shall we not approach nearer the truth, in assigning these different morbid phenomena to the same cause which produces them in persons who are reduced to a state of anæmia by the deprivation of food, light, and wholesome atmosphere?.... Venesections, employed in such cases to combat an irritation which in reality does not exist, invariably produce a marked aggravation of all the symptoms: on the contrary, it frequently happens that by stimulating the nervous system of these chlorotic patients by the physical and moral emotions of matrimony, we produce a more natural complexion and colour of the whole cutaneous surface, thus indicating a corresponding improvement in the process of sanguification, and in proportion as the anæmia disappears under the influence of this new modification of the nervous system, the whole train of diseased action, the difficult respiration, constant sensation of uneasiness and listlessness, impaired digestion, gastralgia, vomiting, tympanitis, and limpid urine, together with all the strange nervous symptoms, which seemed dependent on some organic alterations of the solids, gradually subside and eventually vanish, as a fresh supply of blood is generated in the system."

It will be allowed, after what has been advanced, that chlorosis is not an inflammatory disease. But still—well as this fact is known, and generally as it is admitted—there are certain symptoms which are very common to the disease, as severe headach, pain in the side affecting the breathing, distressing action of the heart, &c., which are often mistaken for and treated as acute inflammatory affections. And we, too, have often "witnessed the very injurious consequences of such mistakes, the practitioner having forgotten, what in female diseases it is peculiarly im-

* Gooch's Lectures, by Skinner, p. 7.

† Pathological Anatomy, by Townsend and West, vol. i. p. 106.

portant to remember, that the severity of the pain and the rapidity of the pulse are generally indications of irritability and excitement, not of inflammation; demanding narcotics, carminatives, and, at the most, counter-irritation, not bleeding, active purgatives, or spare diet." Dr. Ashwell does not touch upon one source of mischievous practice, which we are sure is a very common one, especially on the part of young practitioners. The female friends, and the chlorotic patient herself, almost invariably attribute the symptoms just enumerated to inflammation. They desire to be bled, and they are bled, more from an easy acquiescence with their wish than from a mistaken conviction of the propriety of the practice. Dr. Ashwell is of opinion that "there will be no difficulty in distinguishing chlorosis from chronic affections of the abdominal viscera, especially from hepatic, splenic, and renal disease, or indeed from any morbid state, of which anæmia and pallor are prominent symptoms. The period of life, the peculiar aspect of a chlorotic patient,* and the derangement of menstruation, will ensure a correct diagnosis." We should have worded this paragraph more cautiously, because we believe that there is often some difficulty, at least, in ensuring a correct diagnosis.

The complications of chlorosis, that are briefly described by Dr. Ashwell, are discharges of blood from the stomach by vomiting; chronic derangement of digestion and nutrition; functional affection of the cerebrum and vascular system; ascites and structural change of the lung. Upon the latter complication especially, Dr. Ashwell's remarks are good and practical; they convey a useful lesson to the inexperienced, by showing under what circumstances there is or is not cause for apprehension as to the issue of the disease. Dr. Waller's account of chlorosis is very short and very unsatisfactory.

The *treatment* of chlorosis, to be frequently successful, must be adopted early and most sedulously prosecuted. In the mild form of the disease the remedies must be principally of a constitutional kind; directed to the improvement of the general health, and to the establishment of puberty. If, when these points are gained, the uterine functions are not developed, the delay may probably be attributed to the torpor of the organs of reproduction, and emmenagogues may be employed. In the various complications of chlorosis, it often happens that the affection of the organ principally implicated becomes so prominent, as almost to throw into the shade the original chlorosis and its accompanying amenorrhœa. This is by no means uncommon; but the source of the disease must always be borne in mind. In such cases the treatment will require modification; it will necessarily be less constitutional, and assume more of a topical character. "And lastly, where structural alteration of the lung is threatened or suspected, the management must have especial and almost exclusive reference to this alarming complication, every measure being adopted to avert this greatest of all dangers. Still, even here, it must not be forgotten, that if puberty could be sufficiently developed to allow of even the partial establishment of menstruation, a very formidable feature of the complication would disappear." (Ashwell, p. 22.) We must observe, however, that the probability of the ultimate success of our treatment in such cases will very greatly depend upon the *early* ap-

* Which is depicted to the life in plate iv. of Dr. Marshall Hall's "Commentaries on the Diseases of Females."—Rev.

plication of it. If, as too frequently happens, from negligence on the part of the girl's friends, or of the practitioner, the case is overlooked for some months, the danger will, at least, be very much increased. The treatment of the most common form of chlorosis, namely, that accompanying puberty, may be regarded as the type of the treatment of all the others, embodying the principles which, with greater or less modification, are universally applicable. "It is here, at the very threshold of the disease, when its character is not understood, or when it is treated empirically, that the greatest error is committed. It is viewed as a local, not as a constitutional affection, and many are the individuals who have been sacrificed to the vain and ignorant attempt of prematurely establishing menstruation: mercury, drastic purgatives, and emmenagogues having irretrievably destroyed the constitutional power, and paved the way for phthisical disease." (Ib. p. 23.) Often as this statement has been repeated, it still too frequently remains a dead letter in actual practice. With most other well-informed practitioners, Dr. Ashwell would commence the treatment of chlorosis by special attention to the digestive organs and alimentary canal, regarding the disorder of these as second only in pernicious effect to the peculiarity of constitution already mentioned. Drastic purgatives are very properly condemned. "The best aperients are aloes, rhubarb, the sulphate of soda and manna, and, if an alterative be necessary, the hydrargyrum c. cretâ. Warm-water enemas, too, are very useful in unloading the large intestines. "The compound decoction of aloes with the compound tincture of cardamoms, the compound aloetic pill with the oil of cassia and hyosciamus, and the vinum aloes with the compound tincture of rhubarb are the forms of these medicines I prescribe." Warm clothing and regular exercise are valuable auxiliaries; and nutritious animal diet and mild malt liquor, as soon as the repugnance to them can be conquered, will be beneficial. The improvement of the digestive organs, indicated by return of appetite and the natural and daily evacuation of the bowels, are generally accompanied by alteration of the complexion and by the partial disappearance of the chlorotic hue; rarely by the immediate establishment or return of the catamenial secretion. At this crisis some of the preparations of iron may be exhibited; "and the sulphate (Dr. Ashwell, p. 25) is probably the most efficacious, and possesses more specific properties than any of the rest." When emmenagogues may be employed is an important question. Dr. Ashwell says, with much truth as we know, that when the health is so far improved that there is less pallor, regularity of bowels, and more and better blood, iron itself is often an efficient emmenagogue. "The ammoniacal injection, composed of one drachm of the pure liquor ammoniæ to a pint of milk, daily injected into the vagina has proved efficient in the hospital." When glandular enlargements and other indications of a strumous habit have been associated with chlorosis, the iodide of iron has been extensively tried by Dr. Ashwell, "and with undoubted success." Leeches and mustard cataplasms to the mammæ (expedients which have both been highly spoken of in this disease) he has tried with doubtful effect. Sixteen cases of various forms of chlorosis are detailed at the end of Dr. Ashwell's chapter on this subject.

We must be brief in our notice of Dr. Ashwell's remarks on

*Amenorrhœa.* One point we must notice, because we have known the neglect of it tend to the loss of professional reputation, and the unexpected distress of the patient. We allude to the occasionally mischievous and even fatal effects that result from the simply effected operation of dividing an imperforate hymen. "It must not be forgotten that such patients, with whatever facility the danger may have been removed, and a mere incision is often sufficient, are really exposed to the danger of peritoneal inflammation." We must be careful then not to speak too confidently of the safety of this operation, and we must watch the patient carefully after it has been performed, that we may oppose the possible danger in time. In two cases we have known severe peritoneal inflammation follow the division of imperforate hymen. Many similar instances are on record. Dr. Ramsbotham* mentions several; two of which were fatal; and we give the following brief extract from his lecture, because we are convinced the fact is not generally known. Dr. Waller, for example, says nothing upon the subject. "The inflammation on these occasions seems to have been as severe as puerperal peritonitis itself, and to bear much of the same characters. It cannot be referred to the puncture as a cause, so satisfactorily as to the evacuation of the fluid, and the change consequently effected in the situation and condition of some of the abdominal viscera," (Ramsbotham.)† After offering many good, but we think generally known, remarks upon the limits which ought to be assigned to the use of emmenagogues, Dr. Ashwell gives the following useful caution: "Stimulating fluids have been, as emmenagogues, injected into the uterine cavity; and they may perhaps, by some who have not used them, be yet recommended. Death from peritoneal inflammation has several times followed the practice; and in two instances, occurring under my own eye, fatal results had nearly ensued from most alarming attacks of this most formidable malady." (p. 75.) "Excepting as a means of arresting hemorrhage, I never now inject the uterine cavity." (p. 76.) Dr. Waller, too, (p. 40,) disapproves of the treatment referred to in the above extracts, but without deeming it necessary to give any other than the rather unsatisfactory reason "that he believes all stimulants applied directly to the womb are improper." A fatal case from injecting the uterus is given in our last Number (p. 566); death being supposed to arise from the escape of the injected fluid into the abdominal cavity through the Fallopian tube. Professor Schönlein assures us that an injection of ten grains of aloes in a small quantity of warm water, thrown up the rectum when the menses ought to appear, is more certain than any other emmenagogue. Dr. Ashwell has used this enema twice or three times a day with advantage. Several of the cases which Dr. Ashwell gives of amenorrhœa are rather uncommon, and deserve perusal.

*Vicarious menstruation* is briefly considered by Dr. Ashwell. We may be allowed to relate a certainly uncommon case which occurred in our own practice, as a useful commentary upon Dr. Ashwell's remark, that "he is not aware any case ever terminated fatally." A young lady, who had always enjoyed good health and good spirits, was exposed to some severe and sudden moral emotions, the consequence of which was a total suppression of the menses. After a few months' disappearance of the menses, she threw up from the stomach a small quantity of dark grumous

* Vide Medical Gazette, vol. xvi. p. 327.—Rev. † Loc. cit.

blood mixed with her food, and she continued to do so for many months, the quantity vomited being gradually increased. Between each attack of vomiting there was usually an interval of five or six weeks. Notwithstanding the continued recurrence of these vicarious sanguineous discharges, she remained apparently well in health, nor was her characteristic cheerfulness at all diminished. We gave a decidedly favorable prognosis. The friends, however, became anxious, and a physician of eminence saw the patient with us. He coincided in the favorable opinion we had formed and very confidently stated, of the happy termination of the case; *but in one hour* after we had left this patient, a more than usually severe attack of vomiting came on, and she died faint and exhausted before we could arrive at her residence. Upon dissection the whole mucous surface of the stomach and intestines was found soft, and almost pulpy; and through the whole extent it was evident that blood had been poured forth in large quantities from the capillary vessels. There was no appearance of erosion or lesion of any large blood-vessel. Every other part of the body was perfectly healthy. Let us be cautious then in our prognosis of such cases, and while we admit the very general rule of their safety, let us not forget such occasional exceptions as the above. Gardien speaks with less confidence upon this subject than most other writers; he says,* "Le flux menstruel, par un vice inné, *ou à l'occasion d'une suppression*, peut, par une sorte d'aberration souvent très dangereuse, prendre des voies différentes de l'utérus." The records of medicine teem with cases of "vicarious menstruation," but the most remarkable example we know of is related † by the author we have just quoted.

Dr. Ashwell's description of the various forms of *Dysmenorrhœa* is clear and accurate. We may observe that in some cases of this disease a large quantity of firm coagula is expelled with considerable suffering at every menstrual period; we have known as much as would fill a five or six ounce bottle discharged for several successive months, and a quantity of these coagula are now in our possession. The account of the well-known dysmenorrhœal membrane, which Dr. Ashwell quotes from Dr. Montgomery, is generally correct; but a remarkably splendid preparation of it, which is now before us, rather confirms the statement of Dr. Waller, (page 63,) who calls it "a tough, thick membrane," than that of Dr. Montgomery, who describes it as "thin, flimsy, and very unsubstantial in its texture." In our specimen the whole cavity of the body of the uterus is lined by this morbid product, which closely adheres to the mucous surface of the uterus. We must offer one or two remarks upon the treatment of dysmenorrhœa. "Let the patient," (says Dr. Ashwell, page 109,) "on the first premonition of pain, commence the use of the hot bath at 96 or 98°, and ordinarily remain in it for half or three quarters of an hour," &c. This is good practice in most cases, and will generally be very beneficial; but Dr. Ashwell will, we are sure, allow us to suggest that it is not the best. If, for two or three nights *before the expected menstrual period*, the warm bath is used, or, what is as effectual and much less inconvenient, a piece of flannel, doubled twice or three

* Traité complet d'Accouchemens, tom. i. p. 238, 3me ed.

† Loc. cit. p. 239. (Note.)

times, and soaked in water as hot as the patient can bear it, is wrapped round the lower part of the body, the painful attacks of dysmenorrhœa, even in cases where there had been previously considerable suffering, will frequently be altogether prevented. We have found it necessary, too, in severe cases, to give much larger doses of opium than are recommended by Dr. Ashwell. Dr. Waller, judging from the prescriptions with which he favours us at p. 66, must have been fortunate in meeting with very mild examples of this disease; we think, too, that he will find few practitioners will agree with him in the little confidence he places in the use of the hip-bath, as at least a very useful auxiliary in the treatment.

Upon the subject of *Leucorrhœa*, profuse menstruation, and affections attendant on the decline of the catamenial function, the reader will find in Dr. Ashwell's book concise and instructive descriptions both of the nature and treatment of the maladies referred to. The following statement, the accuracy of which we are fully convinced of from cases which have occurred in our own practice, should inspire more caution than is sometimes exercised by practitioners, upon a question which involves both the honour and happiness of their patients: "One thing is quite true, that, in women of indisputable purity, leucorrhœa is sometimes so acrimonious, as not unfrequently to produce discharge and abrasion in the husband; and, on one or two occasions after abortion, I am almost confident that eruptions and decided ulceration have been among the results of intercourse." (Ashwell, p. 175.)

This first part of Dr. Ashwell's projected work, of which we now terminate our notice, is, upon the whole, creditable to himself, and likely to be useful to those who require the sum and substance of the practical information we at present possess upon the various subjects he treats of. We have no doubt he has drawn his descriptions of disease very faithfully from what he has himself observed; and, as others have done the same before, we meet with much that is contained in other works. Dr. Ashwell proposes to publish in a few months the second part of his work, which will embrace organic diseases; and the third part, shortly thereafter, comprising "the affections of the pregnant and puerperal states."

As Dr. Waller treats in his volume upon many of the diseases described by Dr. Ashwell, we hoped, at the commencement of our notice, to glean some comments from his labours which might be acceptable to our readers. Our attempt, however, which was certainly made with a very impartial spirit, we found to be useless. With regret we are compelled to say that Dr. Waller's sketches of disease are as meager and unsatisfactory as can be well conceived; and we think he has been ill advised in communicating them to the public in their present form. We cannot conclude even this brief notice of his book, without expressing our concern that so respectable a physician should have admitted into his title-page, the superfluous synonymy by which it is disfigured. To professional readers this must at least be useless; and why should Dr. Waller give the ill-natured an opportunity of insinuating that its object was to attract the notice of readers of another class, in the advertising columns of the journals? We ourselves look upon it merely as an example of careless tautology, and condemn it as we do any other literary delinquency; but we are not sure that all our author's readers will judge so leniently.

